# Evaluating the impact of an information-based education and training platform on the incidence, severity, and coping resources status of workplace violence among nurses: a quasi-experimental study

**DOI:** 10.1186/s12912-023-01606-0

**Published:** 2023-11-25

**Authors:** Ying Zhang, Jianzheng Cai, Ziyu Qin, Haifang Wang, Xiuying Hu

**Affiliations:** 1 The First Affiliated Hospital of Soochow University, Suzhou, China; 2 Department of Nursing, Soochow University, Suzhou, China; 3 Department of Nursing, West China Hospital of Sichuan University, Sichuan, China

**Keywords:** Nurses, Workplace violence, Information technology, Education and training, Platform, Questionnaire investigation

## Abstract

**Background:**

Workplace violence among nurses has emerged as a critical issue, posing a significant threat to their occupational safety. Education and training are the primary measures employed to prevent and respond to workplace violence. However, the current approaches have proven ineffective, possibly due to a lack of consideration for the specific needs of clinical nurses. Therefore, it is essential to explore the effectiveness of an informational education and training platform tailored to nurses' requirements. This study aimed to investigate the impact of such a platform on the incidence, severity, and coping resources of WPV in nurses.

**Methods:**

This research was a quasi-experimental study. An information-based education and training platform focused on nurse workplace violence was developed through literature reviews, expert meetings, consultations with software development companies, and a trial run. A tertiary general hospital in Suzhou was selected, in which hospital district A was the intervention group and hospital district B was the control group. A total of 276 nurses were recruited, 140 in the intervention group and 136 in the control group. The nurses' incidence, severity, coping resources status, and evaluation of the application were measured before the intervention and at 1, 3, and 6 months after the intervention.

**Results:**

The overall incidence of workplace violence, verbal aggression, and verbal threat among nurses showed statistically significant differences (*P* < 0.05) for the time effect, while the incidence of physical aggression demonstrated statistically significant differences (*P* < 0.05) for the between-group effect and the time effect. The severity of physical violence among nurses exhibited statistically significant differences (*P* < 0.05) for the between-group effect and time effect, and the severity of psychological violence showed statistically significant differences (*P* < 0.05) for the time effect. Nurses' total coping resources score and dimensions also showed statistically significant differences in terms of group, time, and interaction effects (*P* < 0.001). The evaluation questionnaire for the mobile application indicated usefulness scores of 2 (1, 2); ease of learning scores of 2 (1, 2); ease of use scores of 2 (1, 2); trust scores of 2 (1, 2.75); acceptance score of 1 (1, 2); and satisfaction scores of 2 (1, 2).

**Conclusions:**

Implementing the nurse workplace violence information-based education and training platform proved beneficial in reducing the incidence and severity of workplace violence among nurses and enhancing their coping resources. This outcome suggested the platform's potential for further application and promotion in clinical settings.

## Introduction

Workplace violence (WPV) against healthcare workers has emerged as a significant public health challenge worldwide [1–4]. The American Society for Victims of Crime has conducted an analysis of WPV incidents across seven high-risk occupations, ranking medical personnel fourth in terms of the total average annual number of violent victims (both fatal and nonfatal) [5]. In the UK, NHS reporting data reveals that hospital WPV contributes to 40% of all safety and health incidents [6]. Nurses, in particular, face a high risk of experiencing WPV, as indicated by statistics from the US Department of Labor, showing that nurses are four times more susceptible to such incidents compared to workers in other professions [7, 8]. The prevalence of WPV among nurses ranges from 54.2% to 79.6%, resulting in severe damage to the health and dignity of nurses. Furthermore, it adversely affects the normal functioning of medical organizations, hinders regular employee recruitment, impacts economic interests, and undermines societal trust [9–15]. In response to this concerning issue, various measures have been proposed to prevent and address WPV among nurses, with education and training gradually emerging as the primary recommended approaches. The World Health Organization (WHO) has affirmed in a global bulletin that education and training are crucial initiatives in preventing and controlling WPV. As such, healthcare organizations at all levels should actively develop violence training programs to adequately prepare their staff [16]. In line with this, the US Occupational Safety and Health Administration (OSHA) has issued guidelines for WPV prevention in the healthcare sector, emphasizing the importance of educating and training all staff members on this matter [17].

Previous studies have consistently demonstrated that training plays a pivotal role in enhancing nurses' knowledge, attitudes, self-efficacy, and confidence in effectively dealing with WPV [18, 19]. Similarly, domestic research has also highlighted the positive impact of WPV training on nurses. Those who have received such training exhibit significantly higher levels of confidence in handling aggressive patient behavior and preventing WPV compared to their non-trained counterparts [20, 21]. Consequently, it is evident that educational training contributes to improving nurses' knowledge, attitudes, skills, confidence, and coping abilities when confronted with violence. However, its effectiveness in reducing the incidence of WPV among nurses warrants further investigation. In a meta-analysis conducted by Spelten et al. [22], it is suggested that educational training may not directly lead to a reduction in the incidence of WPV among nurses. In contrast, a study by Fernandes et al. [23] has reported that, surprisingly, the incidence of WPV among nurses is increased slightly after 6 months of training compared to the pre-training period. Furthermore, Laker et al. [24] have found no significant difference in the number and severity of violent incidents before and after the implementation of training.

Heckemann [25] has proposed that the limited effectiveness of WPV education courses in reducing its incidence may be attributed to the broad nature of the training content. Similarly, Tölli et al. [26] have pointed out that the training needs of the study participants may not have been adequately addressed. To address these concerns and cater to the specific WPV education and training needs of nurses, the project team has conducted interviews with 18 nurses who have received WPV training. The findings reveal that nurses prefer training content that focuses on procedural and step-by-step WPV response strategies, akin to "emergency plans". Additionally, the nurses express a desire for greater flexibility and compliance in learning, with an emphasis on utilizing information technology, such as the Internet [27]. To meet the identified WPV education and training needs of nurses, the group has undertaken an analysis of 11 high-risk situations for nurses (Table 1) and devised a standardized prevention and response strategy [28]. Moreover, conventional methods of implementing WPV education and training, such as classroom teaching, offline scenarios, and case studies, though somewhat effective in enhancing communication and coping skills, are found to be resource-intensive, demanding significant human, material, and time resources [29]. However, with the advent of mobile Internet + , access to information has transformed, and the dissemination of knowledge is no longer restricted by time or geographic location. Various forms of information dissemination have emerged, offering new opportunities for education and training. Training through information technology platforms has proven to be a cost-effective, engaging, accessible, and flexible approach, often demonstrating similar or even superior effects to traditional offline training methods [30]. Building upon the demands of nurse education and training, this study presented a nurse WPV information-based education and training platform aimed at delivering precise training to nurses. The platform explored its effectiveness in terms of reducing the incidence and severity of WPV while also enhancing coping resources. To achieve these goals, the platform drew on the previously developed nurse WPV high-risk situations as the foundation for training.

**Table 1:** Nurse WPV high-risk situations

Core Category	Category	Core Category	Category
Preceding states	The patient is an alcoholic or a family member	Environmental elements	The patient is dissatisfied with the general hospital environment
	The patient has an abnormal mental state		The patient is dissatisfied with the hospital system or process
Purpose elements	The patient has repeatedly sought the doctor or nurse without success	Timing elements	The patient is dissatisfied with the nurse's service attitude
	When the patient waits for a long time		When the patient questions the medical expenses
	When the patient's unreasonable request is refused		When the patient refuses to accept the current condition
			When the patient is unsuccessful in the first invasive operation

## Data and methods

### Platform construction and development

**Fig. 1:**
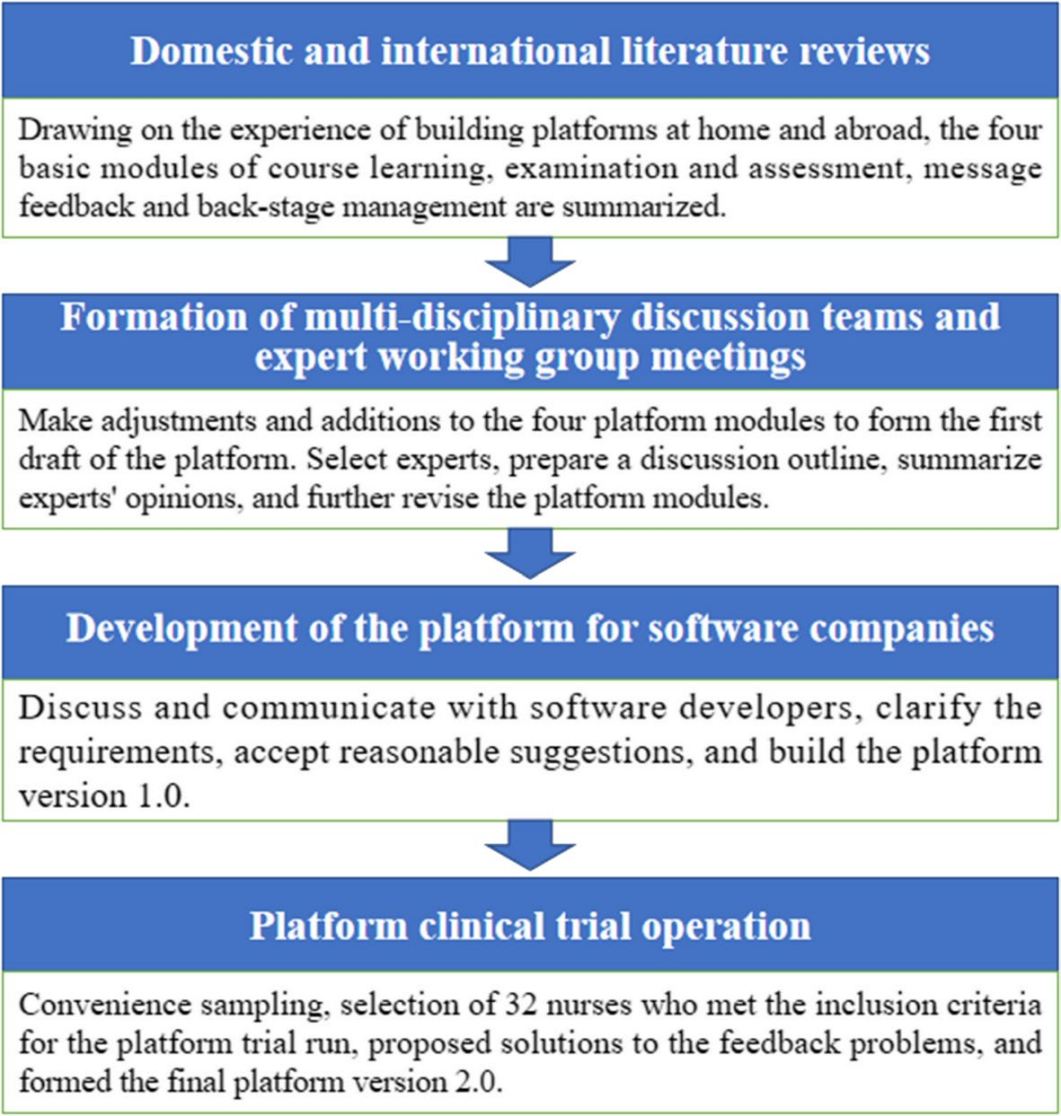
Diagram of the construction process of WPV information-based education and training platform for nurses

**Fig. 2:**
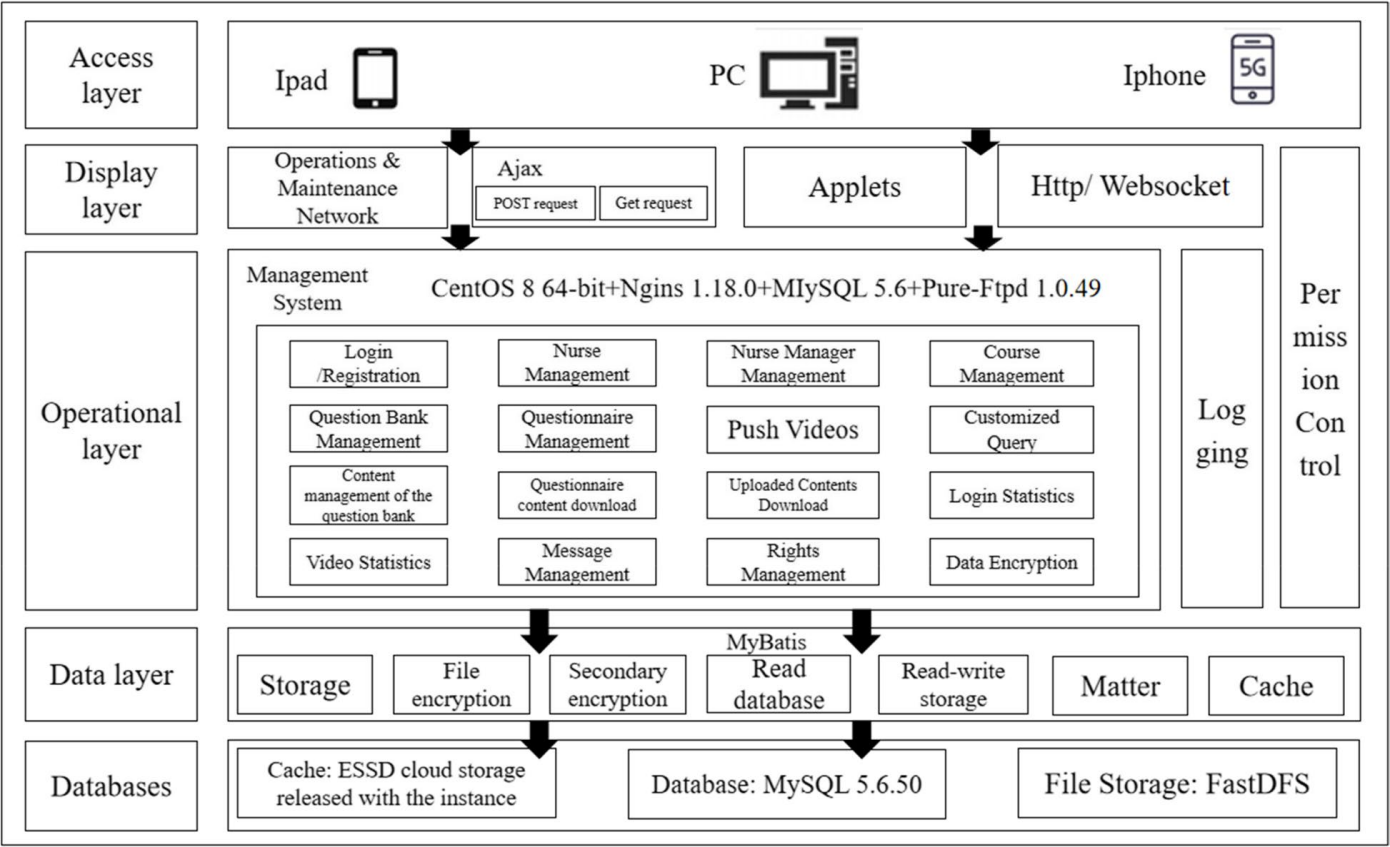
Architecture of WPV information-based education and training platform for nurses

**Fig. 3:**
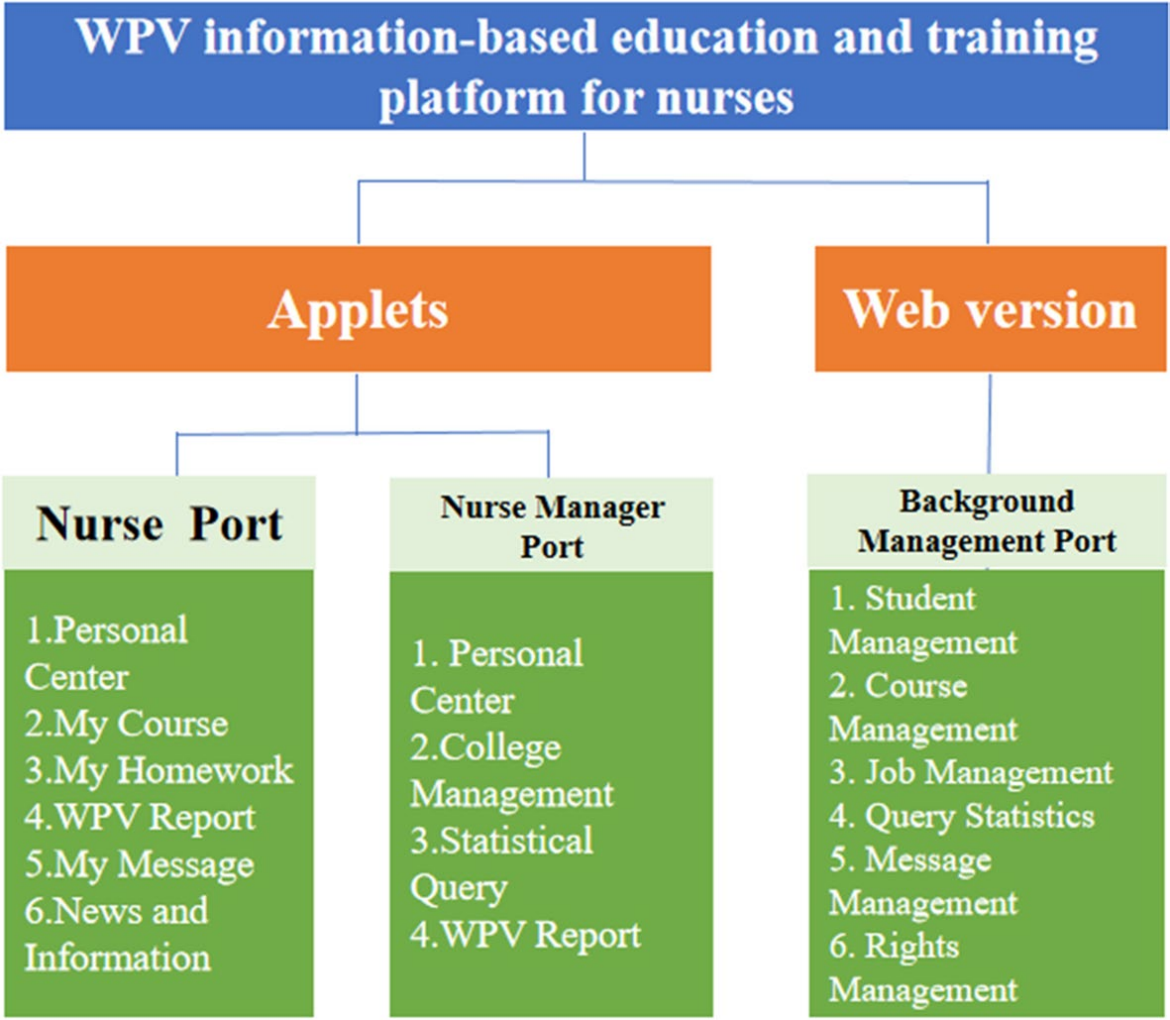
Nurse WPV information-based education and training platform (2.0) version

The platform construction and development took place in Suzhou, China, spanning from July 2020 to April 2021. The process can be outlined as follows. From July to September 2020, a comprehensive review of domestic and international literature was conducted to identify the four fundamental modules of the platform: course learning, examination and assessment, message feedback, and background management. From September to November 2020, a multi-disciplinary research team and an expert working group collaborated to modify and enhance the platform modules. From November 2020 to January 2021, negotiations were carried out with a software development company to define the platform requirements and consider their suggestions, leading to the creation of platform version 1.0. From January to April 2021, a trial operation was conducted among nurses, and based on their feedback, necessary modifications and optimizations were made to develop the final version 2.0 of the platform. The study [31] provides detailed information on the platform's construction and development. Additionally, Fig. 1 illustrates the process of platform construction. Figure 2 depicts the platform architecture, and Fig. 3 showcases platform version 2.0. For a detailed understanding of each module's functions, please refer to Table 2.

**Table 2:** Introduction to the functions of the WPV information-based education and training platform for nurses

Port	Module	Function
Nurse Port	Personal Center	Display personal information of the currently logged-in user, including an avatar, name, login name, gender, hospital, department, contact information, and so on
	My Course	Training videos on WPV concept and classification, causative factors and preventive measures, early warning identification, response, and handling skills, the reporting process, and related medical-legal knowledge
	My Homework	Post-course test: Complete and submit the post-course exercises for each chapter of the WPV course. Nurses who do not reach the passing line need to return to the "My Course" module to study the course again and resubmit the assignments until they pass. ②Evaluation feedback: It is used to collect the comprehensive evaluation data of nurses on the effectiveness of WPV training, including the hospital WPV questionnaire, violence severity rating scale, and hospital WPV coping resources scale
	WPV Report	Nurses experiencing WPV can report it through this module and subsequently view the hospital administration's handling of the situation
	My Message	Leave messages and submit users' confusion, requests, suggestions, and so on
	News and Information	Post links to existing WPV-related national policies, social dynamics, laws, and regulations
Nurse Manager Port	Personal Center	Display personal information of the currently logged-in user, including an avatar, name, login name, gender, hospital, department, contact information, and so on
	College Management	Review of personal information and electronic file management of nurses in the departments under their jurisdiction
	Statistical Query	The head nurse can check the training completion status of nurses in her department through the background, and the nursing department can check the training completion status of nurses in the whole hospital through the background and supervise and remind nurses who have not completed their training tasks
	WPV Report	Provide processing and feedback on WPV events for nurses in their units
Background Management Port	Student Management	Maintenance and updating of nurses' personal information and management of WPV training files
	Course Management	Text, pictures, and videos of training courses are imported or uploaded, and the system automatically reminds nurses of the messages
	Job Management	Course test questions and questionnaires are imported or uploaded, and the system automatically alerts nurses with messages
	Query Statistics	View the training status of all nurses (e.g., number of logins, course progress, length of logins, whether exams were passed, whether questionnaires were completed, and so on)
	Message Management	Includes system operation records and nurse message records
	Rights Management	It includes permission granting and permission verification to realize the permission of different user roles to operate the module functions

### Research on the application effect of information-based education and training platform

#### Study design

This research was a quasi-experimental study to assess the impact of an information-based education and training platform on the incidence, severity, and coping resources of WPV among nurses.

#### Setting and sample

##### Setting

The application effect research of the information platform was conducted from May to December 2021 in Suzhou, China.

##### Sampling

The study subjects were 276 nurses from a tertiary general hospital(The hospital has two districts, A and B) in Suzhou, who were from emergency, outpatient, surgery, internal medicine, ICU and other departments. This is the criteria for inclusion, exclusion and withdrawal of subjects.

##### Inclusion criteria

(1) active nurses in clinical departments that require direct contact with patients in their daily work; (2) registered nurses with nursing practice certificates; (3) 1 year of clinical nursing work; and (4) informed consent and voluntary participation in this study.

##### Exclusion criteria

(1) nurses in regular training or advanced training; and (2) nurses who were in off-duty status during the survey period, such as maternity leave, sick leave, vacation, and so on.

##### Withdrawal criteria

Those who are unable to continue to complete the study due to illness, job change, family change, etc.

#### Recruitment and grouping methods

A tertiary general hospital in Suzhou was selected, in which hospital district A was the intervention group and hospital district B was the control group. The researcher communicated with the hospital administrators, prepared the recruitment announcement for this study, and advertised it through the hospital's official website and corporate WeChat to recruit study participants who met the inclusion criteria for this study. A total of 276 working nurses from Emergency, Outpatient, Surgery, Internal Medicine, ICU and other departments in the hospital who met the inclusion criteria were selected from July to December 2021 as study subjects. A total of 140 nurses who met the inclusion criteria were recruited from hospital district A and 136 nurses who met the inclusion criteria were recruited from hospital district B.

#### Sample size calculation

According to the formula for the sample size of the superiority test [32],

$$N=\frac{({\mathrm{Z}}_{1-\mathrm{\alpha }}+{\mathrm{Z}}_{1-\upbeta })^2}{{(\uppi }_{\mathrm{T}}-{\uppi }_{\mathrm{C}}-\Delta )^2}\times [\frac{{\uppi }_{\mathrm{T}}\left(1-{\uppi }_{\mathrm{T}}\right)}{K}+{\uppi }_{\mathrm{C}}(1-{\uppi }_{\mathrm{C}})]$$

The superiority test is a one-sided test, and the test level α is often taken as 0.025. In this study, β = 0.2, Z_1-α_ = 1.96, Z_1-β_ = 0.842. π_T_ and π_C_ are the incidence of WPV of the intervention and control groups, respectively. The results of the pre-survey showed that the incidence of WPV was 78.5% in the intervention group and 55.6% in the control group. The superiority bound value ∆ was taken as 5%. K is the ratio of intervention and control group, which is set at 1. Considering a 10% sample loss rate, the final sample size for each group was adjusted to 112. Thus, the total sample size for the study will be 224 (112 in the intervention group and 112 in the control group).

#### Intervention program

**Fig. 4:**
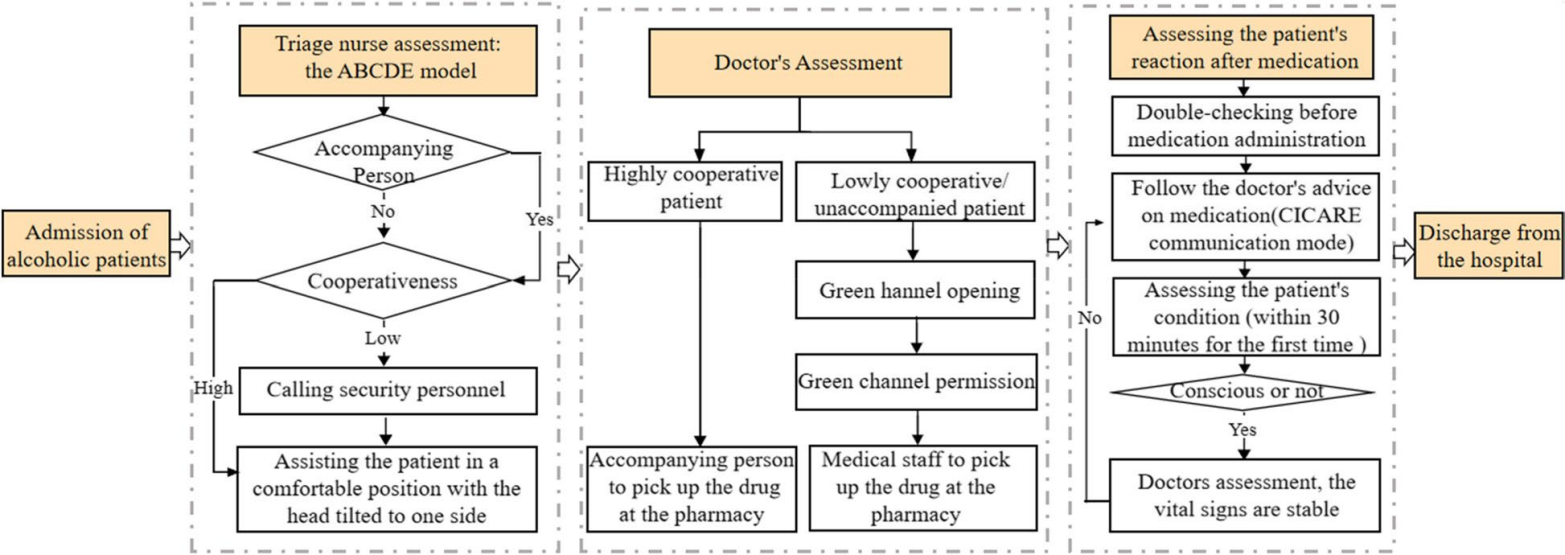
The WPV prevention process for nurses encountering "alcoholic patients"

The intervention strategy of this study was developed based on previous research. Firstly, qualitative interviews were conducted with nurses, focusing on the elements of nurses' WPV high-risk scenarios experienced by nurses. This information was used to create the initial draft of the prevention strategy. Subsequently, an expert focus group discussion team was formed to review and refine the initial draft, resulting in the final version of the prevention strategy. Detailed information on the construction method of the intervention strategy can be found in the study [33]. For example, the WPV prevention strategy for when a nurse encounters an "alcoholic patients" is depicted in Fig. 4.

##### Intervention group

An online intervention using an information-based education and training platform based on prevention and coping strategies for WPV high-risk situational elements.

##### Control group

An offline intervention using prevention and coping strategies for WPV high-risk situational elements. The intervention period was 6 months, and the intervention protocol is shown in Table 3.

**Table 3:** Intervention protocol

Category			Control group	Intervention group
Intervention Objectives			Awareness of WPV prevention, WPV identification skills, WPV response skills, self-protection skills, healthy psychological state, and familiarity with laws and regulations	
Intervention method			Offline training (Classroom lectures and scenario simulations)	Online training (WPV prevention training platform)
Intervention measures	Pre-intervention		Notification of training was issued in advance through relevant departments to inform participants of the time, place, and training contents	Members of the subject group provided unified on-site training and Q&A to nurses in the intervention group and issued the platform user operation manual to enable nurses to master the use of the platform
	Intervention period	Week 1 (8 h)	Basic theoretical knowledge of WPV, and prevention strategies for nurses in high-risk situations of WPV first state	
		Week 2 (8 h)	Prevention strategies for environmental or institutional elements of WPV high-risk situations for nurses	
		Week 3 (8 h)	Prevention strategies for the purpose elements of WPV high-risk situations for nurses	
		Week 4 (8 h)	Prevention strategies for the timing elements of high-risk situations for nurses with WPV	
	Post-intervention		Questionnaires were administered by the researcher at 1, 3, and 6 months after training	Automatic questionnaire push by the platform 1, 3, and 6 months after training

#### Research tools

##### Hospital WPV questionnaire-revised version

In this study, general information about nurses and the frequency of WPV incidents, including verbal aggression, verbal threat, physical aggression, and sexual harassment, were assessed. The questionnaire used in the study was designed by Zuhui Chen from Jinan University [34] and consisted of 16 items organized into 2 dimensions. The design of the questionnaire was based on the WHO's documents and definitions of WPV, ensuring its relevance and compatibility with domestic and international studies [28]. To evaluate the questionnaire's reliability and validity, a pretest was conducted. The pretest reliability of the scale was found to be 0.803, indicating a high level of consistency in the responses. Additionally, the mean validity of the content for each item was 0.916, suggesting that the items effectively measured the intended constructs.

##### Violence severity rating scale

To assess the severity of physical and psychological violence suffered by nurses, a scale was developed by Linhong Zhu [35], which incorporates the Criminal Law of the People's Republic of China and other relevant legal standards. The scale consists of 2 items in 2 dimensions. The severity was assessed by the hospital adverse event management team: (1) Physical violence severity grading: level 0, no physical violence; level 1, physical assault on medical staff without bodily harm; level 2, violence resulting in minor injuries to medical staff; level 3, violence resulting in light injuries to medical staff; level 4, violence resulting in serious injury or death(The standards for minor injuries, light injuries, and serious injuries refer to the《Penal code》). (2) Psychological violence severity grading: level 0, no psychological violence; level l, suffered psychological violence but had no impact; level 2, impact on medical staff such as depression, but still adhering to the work; level 3, obvious impact on the spirit of medical staff, temporarily unable to adhere to the work; level 4, severe anxiety, insomnia, etc., requiring psychological intervention treatment.

##### Hospital WPV coping resources scale

To assess the level of WPV coping resources among nurses, a scale developed by Peixi Wang [36] was utilized. Coping resources refer to a series of comprehensive set of measures employed to alleviate the intensity of the stress response. According to stress theory, coping resources, act as an intermediary variable between stressors and coping outcomes, serving as a buffer for the consequences of stressful events. In other words, higher coping resources score, indicates a greater abundance of coping resources, resulting in reduced negative consequences caused by stressful events [37, 38]. Peixi Wang's scale consists of 4 dimensions and includes 20 items. These dimensions are cognitive ability, anticipatory ability, coping ability, and hospital organizational support for WPV. Nurses were asked to respond to each item using a Likert 6-point scale, with higher score reflecting a higher level of coping resources among nurses. The scale has been widely used in clinical settings and has demonstrated good reliability and validity, as evidenced by a Cronbach’s alpha coefficient of 0.92 in previous surveys [37].

##### Mobile application evaluation questionnaire

To assess the nurses' evaluation of the applet. The questionnaire of this study was formed by referring to the mobile application evaluation questionnaire developed by Fengjiao Dou [39]. There were 11 items in 6 dimensions, namely usefulness (item 1), ease of learning (items 2–3), ease of use (items 4–5), trust (items 6–7), acceptance (items 8–9), and satisfaction (items 10–11), and a Likert 5-point scale was used. Lower scale score indicates higher ratings of mobile applications. The reliability and validity of the questionnaire were good.

#### Data collection

##### Baseline information

Baseline information was gathered from the study participants after providing a detailed explanation of the study's purpose, significance, and methods in hospital training rooms. Informed consent was obtained to ensure the participants' voluntary cooperation in the research. For the intervention group, baseline information was collected when nurses initially logged into the platform. On the other hand, for the control group, the baseline information was distributed to the participants, who filled it in and subsequently handed it back to the researchers during face-to-face interactions. Baseline information includes the demographics of the nurses (age, gender, marital status, whether only child, education, years of experience, title, position, form of employment, and department of work) and the nurse's WPV incidence, WPV severity score, and coping resources score.

##### Post-intervention information

The data were collected at 1, 3, and 6 months after the completion of the training. For the intervention group, data were automatically pushed through the platform, and all questionnaire contents were filled in completely via background settings before submission. As for the control group, data were collected through face-to-face interactions with the group members in hospital training rooms. Nurse WPV incidence, WPV severity score, and coping resources score were collected at months 1, 3, and 6 of the intervention, and a mobile application evaluation questionnaire score was added at month 6.

#### Data analysis

SPSS 21.0 was utilized for statistical analysis, and a significance level of *P* < 0.05 was considered to indicate a statistically significant difference. Qualitative data were described using frequency and component ratio (%). Measurement data that adhered to a normal distribution were described by mean ± standard deviation ($\overline{X }$±*S*), while non-normally distributed data were represented by the median and quartiles(P25, P75). Specific analysis methods were as follows. Normality and chi-square tests were employed to assess the data using the Shapiro–Wilk test. General information characteristics of the nurses, WPV incidence, WPV severity, WPV coping resources score, and mobile application score were described using mean ± standard deviation, median and quartiles, and frequency (composition ratio). The chi-square test was used to compare the general information and WPV incidence among the nurses. The rank sum test was employed to compare the severity of WPV experienced by the nurses. Generalized Estimating Equation (GEE) was used to analyze changes in WPV incidence, WPV severity, and coping resources over time.

#### Quality control

##### Before the start of the study

After training and guidance on platform operation, nurses in the intervention group were able to use the wechat application for learning without any problems.

##### Platform use and data collection phase

Nurses in the control group distributed and collected questionnaires face to face, and gave feedback and solved any problems in the questionnaire on the spot. Nurses in the intervention group need to complete the training within 4 weeks and could not complete the training by pulling the progress bar. Researchers could monitor the use of platform in real time through the background. After the training is completed, the platform will automatically send a wechat notification to the nurse to fill in the questionnaire. The platform has a message function, nurses have any questions can be timely feedback, researchers will promptly review and reply.

#### Ethical considerations

The study was approved by the Ethical Review Committee of the First Affiliated Hospital of Soochow University with approval number 2018062. Before participating in the survey, all participants provided their informed consent for enrollment. All methods used in the study were carried out in accordance with relevant guidelines and regulations. Participants are enrolled in this study solely on their own volition, and the participant has the right to refuse or withdraw from this study at any time, which will not affect any of his medical treatment or rights, nor will he be discriminated against by medical staff.

## Results

### General information about the study subjects

The study included a total of 276 nurses, with 140 assigned to the intervention group and 136 to the control group, all of whom did not experience shedding. Baseline information, including gender, age, marital status, working years, work department, title, and position, was collected for both groups of nurses. Statistical analysis showed no significant difference between the two groups in terms of these baseline characteristics (*P* > 0.05). The general information for both groups is presented in Table 4.

**Table 4:** Comparison of general information of study subjects (%)

Item	Category	Intervention group (*n* = 140)	Control group (*n* = 136)	^χ2^/Z	*P*
Age	< 30	61(43.57)	48(29.32)	-1.342^a^	0.180
	30 ~ 39	49(35.00)	52(49.26)		
	40 ~ 49	22(15.71)	30(25.00)		
	≥ 50	8(5.71)	6(4.41)		
Gender	Male	12(8.57)	6(4.41)	1.958	0.162
	Female	128(91.43)	130(95.59)		
Marital status	Married	99(70.71)	110(83.82)	4.335	0.114
	Unmarried	37(26.43)	22(13.24)		
	Others	4(2.86)	4(2.94)		
Only child	Yes	47(33.57)	55(40.44)	1.397	0.237
	No	93(66.43)	81(59.56)		
Education level	College and below	8(5.71)	14(10.29)	-1.579^a^	0.114
	Bachelor's degree	128(91.43)	120(88.24)		
	Master's degree and above	4(2.86)	2(1.47)		
Working years	≤ 5	29(20.71)	24(5.88)	-0.828^a^	0.408
	6 ~ 10	62(44.29)	60(27.94)		
	11 ~ 15	24(17.14)	22(41.18)		
	≥ 16	25(17.86)	30(25.00)		
Title	Nurse	15(10.71)	20(1.47)	-0.076^a^	0.940
	Nurse Practitioner	68(48.57)	56(38.24)		
	Nurse Supervisor	40(28.57)	46(50.00)		
	Deputy Chief Nursing Officer and above	17(12.14)	14(10.29)		
Position	Nurse	132(94.29)	127(93.38)	-0.312^a^	0.755
	Head Nurse and above	8(5.71)	9(6.62)		
Employment form	Staffed employees	31(22.14)	41(52.21)	2.292	0.130
	Contract employees	109(77.86)	95(4.79)		
Work department	Emergency	30(21.43)	29(21.32)	4.758	0.446
	Outpatient	20(14.29)	30(22.06)		
	Surgery	34(24.29)	33(24.26)		
	Internal Medicine	28(20.00)	25(18.38)		
	ICU	20(14.29)	11(8.09)		
	Others	8(5.71)	8(5.89)		

"^a^"using Mann–Whitney U test

### Evaluation of the application effect of WPV information-based education and training platform for nurses

#### Incidence of WPV in nurses

The incidence of WPV among nurses was treated as qualitative data, and for data analysis, GEE was used. As shown in Table 5, the overall incidence of WPV, verbal aggression, and verbal threat among nurses demonstrated a statistically significant time effect (*P* < 0.05). Additionally, the incidence of physical aggression showed statistical significance in terms of both the between-group effect and the time effect (*P* < 0.05). Further analysis of the intervention group revealed that the differences in the overall incidence of WPV, verbal aggression, and verbal threat among nurses were statistically significant (*P* < 0.05) when comparing the pre-intervention period with 3 and 6 months post-intervention, as well as 1-month post-intervention with 3 and 6 months post-intervention. Moreover, the incidence of physical aggression among nurses in the intervention group showed statistically significant differences (*P* < 0.05) when comparing the pre-intervention period with 1, 3, and 6 months post-intervention. In the control group, the differences in the overall incidence of WPV and verbal aggression were statistically significant (*P* < 0.05) between 1 month after the intervention and 6 months after the intervention. Additionally, the incidence of physical aggression showed a statistically significant difference (*P* < 0.05) between the pre-intervention period and 6 months after the intervention. Detailed results are presented in Table 6.

**Table 5:** Comparison of the incidence of WPV between the two groups of nurses (%)

Category	Time point	Intervention group (*n* = 140)	Control group (*n* = 136)	Between-group effect		Time effect		Interaction effect	
				*Wald χ*^2^	*P*	*Wald χ*^2^	*P*	*Wald χ*^2^	*P*
Overall	T_0_	84(60.00)	82(60.29)	2.158	0.142	32.938	< 0.001	6.517	0.089
	T_1_	96(68.57)	92(67.65)						
	T_2_	67(47.86)	82(60.29)						
	T_3_	61(43.57)	76(55.89)						
Verbal aggression	T_0_	83(59.29)	81(59.56)	2.733	0.098	32.992	< 0.001	4.667	0.198
	T_1_	92(65.71)	91(66.91)						
	T_2_	65(46.63)	80(58.82)						
	T_3_	60(42.86)	75(55.15)						
Verbal threat	T_0_	60(42.86)	58(42.65)	2.799	0.094	32.205	< 0.001	7.270	0.064
	T_1_	75(53.57)	69(46.32)						
	T_2_	42(30.00)	57(41.91)						
	T_3_	38(27.14)	53(38.97)						
Verbal sexual harassment	T_0_	22(15.71)	20(14.71)	1.171	0.279	0.718	0.869	0.167	0.983
	T_1_	23(16.43)	19(13.97)						
	T_2_	27(19.29)	21(15.44)						
	T_3_	24(17.14)	20(14.71)						
Physical aggression	T_0_	41(29.29)	40(29.41)	5.222	0.022	21.770	< 0.001	4.174	0.243
	T_1_	23(16.43)	28(20.59)						
	T_2_	16(12.86)	27(19.85)						
	T_3_	14(10.00)	25(18.38)						
Physical sexual harassment	T_0_	7(5.00)	7(5.15)	0.116	0.733	1.070	0.784	1.578	0.664
	T_1_	6(4.29)	4(2.94)						
	T_2_	5(3.57)	5(3.68)						
	T_3_	4(2.86)	8(5.89)						

T0: baseline; T1: 1 month after training; T2: 3 months after training; T3: 6 months after training

**Table 6:** Two-by-two comparison of the incidence of WPV at different time points within the group of nurses in both groups

Group	Category	Time point	^χ2^	*P*
Intervention group	Overall	T_0_-T_1_	1.891	0.169
		T_0_-T_2_	4.154	0.042
		T_0_-T_3_	7.567	0.006
		T_1_-T_2_	12.348	< 0.001
		T_1_-T_3_	17.762	< 0.001
		T_2_-T_3_	0.518	0.472
	Verbal aggression	T_0_-T_1_	1.234	0.267
		T_0_-T_2_	4.644	0.031
		T_0_-T_3_	7.561	0.006
		T_1_-T_2_	10.570	0.001
		T_1_-T_3_	14.737	< 0.001
		T_2_-T_3_	0.361	0.548
	Verbal threat	T_0_-T_1_	3.218	0.073
		T_0_-T_2_	4.997	0.025
		T_0_-T_3_	7.598	0.006
		T_1_-T_2_	15.989	< 0.001
		T_1_-T_3_	20.313	< 0.001
		T_2_-T_3_	0.280	0.597
	Verbal sex harassment	T_0_-T_1_	0.026	0.871
		T_0_-T_2_	0.618	0.432
		T_0_-T_3_	0.104	0.747
		T_1_-T_2_	0.390	0.533
		T_1_-T_3_	0.026	0.873
		T_2_-T_3_	0.216	0.642
	Physical aggression	T_0_-T_1_	6.563	0.010
		T_0_-T_2_	13.768	< 0.001
		T_0_-T_3_	16.495	< 0.001
		T_1_-T_2_	1.460	0.227
		T_1_-T_3_	2.523	0.112
		T_2_-T_3_	0.149	0.699
	Physical sex harassment	T_0_-T_1_	0.081	0.776
		T_0_-T_2_	0.348	0.555
		T_0_-T_3_	0.379	0.538
		T_1_-T_2_	0.095	0.758
		T_1_-T_3_	0.104	0.747
		T_2_-T_3_	0.115	0.735
Control group	Overall	T_0_-T_1_	1.595	0.207
		T_0_-T_2_	0.000	1.000
		T_0_-T_3_	0.544	0.461
		T_1_-T_2_	1.595	0.207
		T_1_-T_3_	3.985	0.046
		T_2_-T_3_	0.544	0.461
	Verbal aggression	T_0_-T_1_	1.581	0.209
		T_0_-T_2_	0.015	0.902
		T_0_-T_3_	0.541	0.462
		T_1_-T_2_	1.906	0.167
		T_1_-T_3_	3.957	0.047
		T_2_-T_3_	0.375	0.540
	Verbal threat	T_0_-T_1_	1.787	0.181
		T_0_-T_2_	0.015	0.902
		T_0_-T_3_	0.381	0.537
		T_1_-T_2_	2.129	0.145
		T_1_-T_3_	3.805	0.051
		T_2_-T_3_	0.244	0.621
	Verbal sex harassment	T_0_-T_1_	0.030	0.863
		T_0_-T_2_	0.029	0.865
		T_0_-T_3_	0.000	1.000
		T_1_-T_2_	0.117	0.732
		T_1_-T_3_	0.030	0.863
		T_2_-T_3_	0.029	0.865
	Physical aggression	T_0_-T_1_	2.824	0.093
		T_0_-T_2_	0.163	0.686
		T_0_-T_3_	4.548	0.033
		T_1_-T_2_	0.023	0.880
		T_1_-T_3_	0.211	0.646
		T_2_-T_3_	0.095	0.758
	Physical sex harassment	T_0_-T_1_	0.379	0.538
		T_0_-T_2_	0.087	0.768
		T_0_-T_3_	0.071	0.791
		T_1_-T_2_	0.000	1.000
		T_1_-T_3_	0.785	0.376
		T_2_-T_3_	0.323	0.570

T0: baseline; T1: 1 month after training; T2: 3 months after training; T3: 6 months after training

#### Comparison of the severity of WPV in nurses

The severity of WPV among nurses was considered qualitative data, and for data analysis, the GEE was utilized. As shown in Table 7, the severity of nurses' physical violence demonstrated statistical significance for both the between-group effect and the time effect (*P* < 0.05). Additionally, the severity of psychological violence was statistically significant for the time effect (*P* < 0.05), as displayed in Table 8. Further analysis of the intervention group indicated that the differences in the severity of physical violence among nurses were statistically significant (*P* < 0.05) when comparing the pre-intervention period with 1, 3, and 6 months after the intervention. Moreover, the severity of psychological violence among nurses in the intervention group showed statistically significant differences (*P* < 0.05) when comparing the pre-intervention period with 3 and 6 months after the intervention, as well as 1 month after the intervention. In the control group, the severity of physical violence showed statistically significant differences (*P* < 0.05) between the pre-intervention period and 6 months after the intervention. Additionally, the severity of psychological violence exhibited statistically significant differences (*P* < 0.05) between 1 month after the intervention and 6 months after the intervention. Detailed results can be found in Table 9.

**Table 7:** Comparison of workplace physical violence severity between two groups of nurses (%)

Group	Time point	Physical violence				
		0	1	2	3	4
Control group (*n* = 136)	T_0_	96(70.59)	36(26.47)	3(2.21)	1(0.74)	0(0.00)
	T_1_	108(79.41)	25(18.38)	3(2.21)	0(0.00)	0(0.00)
	T_2_	109(80.15)	24(17,65)	2(1.47)	1(0.74)	0(0.00)
	T_3_	111(81.62)	23(16.91)	2(1.47)	0(0.00)	0(0.00)
Intervention group (*n* = 140)	T_0_	99(70.71)	36(25.71)	3(2.14)	2(1.43)	0(0.00)
	T_1_	117(83.57)	20(14.29)	2(1.43)	1(0.71)	0(0.00)
	T_2_	124(88.57)	15(10.71)	1(0.71)	0(0.00)	0(0.00)
	T_3_	126(90.00)	14(10.00)	0(0.00)	0(0.00)	0(0.00)
Between-group effect	*Wald* χ^2^			4.757		
	*P*			0.029		
Time effect	*Wald* χ^2^			23.966		
	*P*			< 0.001		
Interaction effect	*Wald* χ^2^			4.501		
	*P*			0.212		

T0: baseline; T1: 1 month after training; T2: 3 months after training; T3: 6 months after training

**Table 8:** Comparison of workplace psychological violence severity between two groups of nurses (%)

Group	Time point	Psychological violence				
		0	1	2	3	4
Control group (*n* = 136)	T_0_	56(41.18)	30(22.06)	48(35.29)	2(1.47)	0(0.00)
	T_1_	45(33.09)	44(32.35)	45(33.09)	2(1.74)	0(0.00)
	T_2_	56(41.18)	39(28.68)	40(29.41)	1(0.74)	0(0.00)
	T_3_	62(45.59)	36(26.47)	38(28.68)	0(0.00)	0(0.00)
Intervention group (*n* = 140)	T_0_	57(40.71)	30(21.43)	51(36.43)	2(1.43)	0(0.00)
	T_1_	48(34.29)	45(32.14)	47(33.57)	0(0.00)	0(0.00)
	T_2_	75(53.57)	31(22.14)	34(24.29)	0(0.00)	0(0.00)
	T_3_	80(57.14)	27(19.29)	32(22.86)	1(0.71)	0(0.00)
Between-group effect	*Wald* χ^2^			2.320		
	*P*			0.128		
Time effect	*Wald* χ^2^			27.446		
	*P*			< 0.001		
Interaction effect	*Wald* χ^2^			3.470		
	*P*			0.325		

T0: baseline; T1: 1 month after training; T2: 3 months after training; T3: 6 months after training

**Table 9:** Two-by-two comparison of the severity of WPV at different time points in the nurses' in-group groups of both groups

Group	Category	Time point	*Z*	*P*
Intervention group	Physical violence	T_0_-T_1_	-2.541	0.011
		T_0_-T_2_	-3.738	< 0.001
		T_0_-T_3_	-4.121	< 0.001
		T_1_-T_2_	-1.237	0.216
		T_1_-T_3_	-1.637	0.102
		T_2_-T_3_	-0.405	0.686
	Psychological violence	T_0_-T_1_	-0.150	0.881
		T_0_-T_2_	-2.560	0.010
		T_0_-T_3_	-2.944	0.003
		T_1_-T_2_	-2.931	0.003
		T_1_-T_3_	-3.346	0.001
		T_2_-T_3_	-0.461	0.645
Control group	Physical violence	T_0_-T_1_	-1.666	0.096
		T_0_-T_2_	-1.806	0.071
		T_0_-T_3_	-2.145	0.032
		T_1_-T_2_	-0.144	0.886
		T_1_-T_3_	-0.479	0.632
		T_2_-T_3_	-0.333	0.739
	Psychological violence	T_0_-T_1_	-0.582	0.560
		T_0_-T_2_	-0.618	0.537
		T_0_-T_3_	-1.281	0.200
		T_1_-T_2_	-1.275	0.202
		T_1_-T_3_	-1.973	0.049
		T_2_-T_3_	-0.699	0.484

T0: baseline; T1: 1 month after training; T2: 3 months after training; T3: 6 months after training

#### Comparison of nurse WPV coping resources score

The Shapiro–Wilk normality test revealed that the WPV coping resources score of nurses in both groups did not follow a normal distribution at all time points. Therefore, GEE was employed for data analysis. As shown in Table 10, the nurses' cognitive ability, anticipatory ability, coping ability, organizational support, and total coping resources score displayed statistical significance in terms of between-group effects, time effects, and interaction effects (*P* < 0.001). Furthermore, the differences between the cognitive ability, anticipatory ability, coping ability, organizational support, and total coping resources score of nurses in the intervention group and the control group at 3 and 6 months after the intervention were statistically significant (*P* < 0.05), as shown in Table 11. Additionally, the differences in cognitive ability, anticipatory ability, coping ability, and total coping resources score among nurses in the intervention group were statistically significant (*P* < 0.05) when comparing pre-intervention with 1, 3, and 6 months after the intervention, as well as 1 month after the intervention with 3 and 6 months after the intervention. Moreover, the differences in organizational support of nurses in the intervention group were statistically significant (*P* < 0.05) when comparing pre-intervention with 1, 3, and 6 months after the intervention, as well as 1 month after the intervention with 3 and 6 months after the intervention. On the other hand, the differences in total coping resources score and coping ability of nurses in the control group were statistically significant (*P* < 0.05) when comparing pre-intervention with 1 and 3 months after the intervention, 1 month after the intervention with 3 and 6 months after the intervention, and 3 months after the intervention with 6 months after the intervention. Finally, the differences in cognitive ability and anticipatory ability among nurses in the control group were statistically significant (*P* < 0.05) when comparing pre-intervention with 1 month after the intervention, as well as 1 month after the intervention with 3 and 6 months after the intervention. Furthermore, the differences in organizational support were statistically significant (*P* < 0.05) when comparing pre-intervention with 1 and 6 months after the intervention, as well as 1 month post-intervention with 3 and 6 months post-intervention, as detailed in Table 12.

**Table 10:** Comparison of WPV coping resources between the two groups of nurses

Category	Time Point	Intervention group (*n* = 140)	Control group (*n* = 136)	Between-group effect		Time effect		Interaction effect	
				*Wald* χ^2^	*P*	*Wald* χ^2^	*P*	*Wald* χ^2^	*P*
Total coping resources score	T_0_	66.00(59.00,82.00)	66.00(58.00,77.00)	49.589	< 0.001	64.244	< 0.001	54.651	< 0.001
	T_1_	78.00(64.00,90.75)	75.00(64.00,85.75)						
	T_2_	80.00(67.00,96.00)	71.50(62.25,79.00)						
	T_3_	80.00(75.00,98.00)	69.00(62.00,75.00)						
Cognitive ability	T_0_	20.50(18.00,24.75)	20.00(18.00,24.00)	48.665	< 0.001	37.941	< 0.001	37.019	< 0.001
	T_1_	24.00(19.25,29.00)	22.75(18.75,27.00)						
	T_2_	24.00(20.00,30.00)	21.50(18.00,24.00)						
	T_3_	24.00(23.00,30.00)	20.00(18.00,24.00)						
Anticipatory ability	T_0_	15.50(14.00,20.00)	15.00(14.00,19.00)	40.146	< 0.001	45.756	< 0.001	30.825	< 0.001
	T_1_	20.00(15.00,22.00)	17.50(15.00,21.00)						
	T_2_	20.00(15.00,24.00)	17.00(15.00,19.00)						
	T_3_	20.00(17.00,25.00)	16.00(15.00,19.00)						
Coping ability	T_0_	23.00(21.00,29.75)	23.00(20.00,27.75)	35.589	< 0.001	68.185	< 0.001	52.940	< 0.001
	T_1_	27.00(22.00,31.00)	26.00(21.00,30.75)						
	T_2_	28.00(23.00,34.00)	26.00(21.00,28.00)						
	T_3_	28.00(26.00,35.00)	24.00(21.00,28.00)						
Organizational support	T_0_	7.00(6.00,10.00)	7.00(6.00,8.00)	35.801	< 0.001	61.818	< 0.001	56.726	< 0.001
	T_1_	8.00(7.00,10.00)	8.00(7.00,10.00)						
	T_2_	9.00(8.00,10.00)	8.00(6.00,8.75)						
	T_3_	10.00(8.00,10.00)	8.00(7.00,8.00)						

T0: baseline; T1: 1 month after training; T2: 3 months after training; T3: 6 months after training

**Table 11:** Comparison of WPV coping resources score between two groups of nurses at the same time point

Category	Time point	Group	Mean	Standard error	*P*	95% confidence interval of the difference	
						Upper	Lower
Total coping resources score	T_0_	T-C	2.738	2.037	0.179	-1.254	6.730
	T_1_	T-C	2.143	1.930	0.267	-1.641	5.926
	T_2_	T-C	11.169	1.667	< 0.001	7.903	14.436
	T_3_	T-C	16.529	1.420	< 0.001	13.746	19.312
Cognitive ability	T_0_	T-C	1.025	0.673	0.127	-0.293	2.344
	T_1_	T-C	0.652	0612	0.286	-0.546	1.851
	T_2_	T-C	3.523	0.590	< 0.001	2.367	4.679
	T_3_	T-C	4.857	0.511	< 0.001	3.856	5.858
Anticipatory ability	T_0_	T-C	0.941	0.583	0.107	-0.202	2.084
	T_1_	T-C	0.723	0.539	0.180	-0.334	1.780
	T_2_	T-C	2.822	0.470	< 0.001	1.901	3.743
	T_3_	T-C	3.913	0.405	< 0.001	3.120	4.705
Coping ability	T_0_	T-C	0.616	0.764	0.420	-0.882	2.114
	T_1_	T-C	0.554	0.720	0.442	-0.857	1.965
	T_2_	T-C	3.492	0.691	< 0.001	2.139	4.846
	T_3_	T-C	6.121	0.636	< 0.001	4.874	7.368
Organizational support	T_0_	T-C	0.156	0.258	0.544	-0.349	0.662
	T_1_	T-C	0.213	0.228	0.351	-0.234	0.661
	T_2_	T-C	1.332	0.188	< 0.001	0.963	1.700
	T_3_	T-C	1.639	0.147	< 0.001	1.350	1.927

T0: baseline; T1: 1-month post-training; T2: 3 months post-training; T3: 6 months post-training; T: intervention group; C: control group

**Table 12:** Two-by-two comparison of WPV coping resources score at different time points within the two nurse groups

Group	Category	Time point	Mean	Standard error	*P*	95% confidence interval of the difference	
						Lower	Upper
Intervention group	Total coping resources score	T_0_-T_1_	-8.243	2.127	< 0.001	-12.411	-4.075
		T_0_-T_2_	-11.336	2.102	< 0.001	-15.279	-7.392
		T_0_-T_3_	-14.357	1.904	< 0.001	-18.089	-10.625
		T_1_-T_2_	-3.093	1.873	0.099	-6.764	0.578
		T_1_-T_3_	-6.114	2.021	0.002	-10.075	-2.154
		T_2_-T_3_	-3.021	1.839	0.100	-6.626	0.583
	Cognitive ability	T_0_-T_1_	-2.186	0.721	0.002	-3.598	-0.774
		T_0_-T_2_	-2.836	0.665	< 0.001	-4.139	-1.532
		T_0_-T_3_	-3.479	0.614	< 0.001	-4.682	-2.275
		T_1_-T_2_	-0.650	0.601	0.279	-1.828	0.528
		T_1_-T_3_	-1.293	0.605	0.033	-2.479	-0.107
		T_2_-T_3_	-0.643	0.571	0.260	-1.762	0.476
	Anticipatory ability	T_0_-T_1_	-1.893	0.605	0.002	-3.079	-0.706
		T_0_-T_2_	-2.529	0.584	< 0.001	-3.674	-1.383
		T_0_-T_3_	-3.450	0.566	< 0.001	-4.558	-2.342
		T_1_-T_2_	-0.636	0.517	0.219	-1.649	0.378
		T_1_-T_3_	-1.557	0.569	0.006	-2.672	-0.443
		T_2_-T_3_	-0.921	0.527	0.080	-1.954	0.111
	Coping ability	T_0_-T_1_	-3.100	0.734	< 0.001	-4.538	-1.662
		T_0_-T_2_	-4.443	0.733	< 0.001	-5.880	-3.005
		T_0_-T_3_	-5.579	0.724	< 0.001	-6.998	-4.160
		T_1_-T_2_	-1.343	0.707	0.057	-2.728	0.043
		T_1_-T_3_	-2.479	0.799	0.002	-4.045	-0.912
		T_2_-T_3_	-1.136	0.736	0.123	-2.579	0.308
	Organizational support	T_0_-T_1_	-1.064	0.267	< 0.001	-1.587	-0.542
		T_0_-T_2_	-1.529	0.235	< 0.001	-1.989	-1.068
		T_0_-T_3_	-1.850	0.226	< 0.001	-2.294	-1.406
		T_1_-T_2_	-0.464	0.209	0.027	-0.875	-0.054
		T_1_-T_3_	-0.786	0.213	< 0.001	-1.202	-0.369
		T_2_-T_3_	-0.321	0.182	0.078	-0.679	0.036
Control group	Total coping resources score	T_0_-T_1_	-8.838	1.773	< 0.001	-12.314	-5.363
		T_0_-T_2_	-2.904	1.360	0.033	-5.569	-2.239
		T_0_-T_3_	-0.566	1.396	0.685	-3.302	2.169
		T_1_-T_2_	5.934	1.539	< 0.001	2.917	8.951
		T_1_-T_3_	8.272	1.444	< 0.001	5.441	11.103
		T_2_-T_3_	2.338	1.100	0.034	0.182	4.494
	Cognitive ability	T_0_-T_1_	-2.559	0.572	< 0.001	-3.679	-1.439
		T_0_-T_2_	-0.338	0.465	0.467	-1.250	0.573
		T_0_-T_3_	0.353	0.522	0.499	-0.669	1.375
		T_1_-T_2_	2.221	0.535	< 0.001	1.171	3.270
		T_1_-T_3_	2.912	0.539	< 0.001	1.855	3.968
		T_2_-T_3_	0.691	0.456	0.130	-0.203	1.586
	Anticipatory ability	T_0_-T_1_	-2.110	0.509	< 0.001	-3.108	-1.112
		T_0_-T_2_	-0.647	0.413	0.117	-1.456	0.162
		T_0_-T_3_	-0.478	0.398	0.230	-1.259	0.303
		T_1_-T_2_	1.463	0.455	0.001	0.572	2.355
		T_1_-T_3_	1.632	0.412	< 0.001	0.824	2.441
		T_2_-T_3_	0.169	0.310	0.585	-0.438	0.777
	Coping ability	T_0_-T_1_	-3.162	0.710	< 0.001	-4.553	-1.770
		T_0_-T_2_	-1.566	0.612	0.011	-2.766	-0.367
		T_0_-T_3_	-0.074	0.571	0.898	-1.192	1.045
		T_1_-T_2_	1.596	0.654	0.015	0.313	2.878
		T_1_-T_3_	3.088	0.607	< 0.001	1.898	4.279
		T_2_-T_3_	1.493	0.601	0.013	0.315	2.670
	Organizational support	T_0_-T_1_	-1.007	0.205	< 0.001	-1.410	-0.605
		T_0_-T_2_	-0.353	0.215	0.100	-0.774	0.068
		T_0_-T_3_	-0.368	0.186	0.048	-0.732	-0.003
		T_1_-T_2_	0.654	0.196	0.001	0.270	1.039
		T_1_-T_3_	0.640	0.160	< 0.001	0.326	0.954
		T_2_-T_3_	-0.015	0.153	0.924	-0.315	0.286

T0: baseline; T1: 1 month after training; T2: 3 months after training; T3: 6 months after training

#### Mobile application evaluation questionnaire score

The mobile application evaluation questionnaire consisted of the following 6 main dimensions: usefulness scores of 2 (1, 2); ease of learning scores of 2 (1, 2); ease of use scores of 2 (1, 2); trust scores of 2 (1, 2.75); acceptance score of 1 (1, 2); and satisfaction scores of 2 (1, 2). The detailed scores for each dimension and item are shown in Table 13.

**Table 13:** Scores for each entry of the mobile application evaluation form [n(%)]

Dimension	Item	Strongly agree	Agree	No opinion	Disagree	Strongly disagree
Usefulness	Using this mobile application is helpful to you	53(37.9)	83(59.3)	4(2.9)	0(0.0)	0(0.0)
Ease of learning	You can learn how to use the mobile application very quickly	54(38.6)	82(58.6)	4(2.9)	0(0.0)	0(0.0)
	You quickly become proficient with the mobile application	53(37.9)	82(58.6)	5(3.6)	0(0.0)	0(0.0)
Ease of use	You find it easy to operate the mobile application	64(45.7)	72(51.4)	3(2.1)	1(0.7)	0(0.0)
	You find the interface of the mobile application easy to switch from one function to another	59(42.1)	72(51.4)	5(3.6)	4(2.9)	0(0.0)
Trust	You won't worry about information leakage with this mobile app	55(39.3)	50(35.7)	16(11.4)	19(13.6)	0(0.0)
	You feel that the hospital can keep your information safe	56(40.0)	49(35.0)	17(12.1)	18(12.9)	0(0.0)
Acceptance	You are happy to use the mobile application	83(59.3)	55(39.3)	2(1.4)	0(0.0)	0(0.0)
	You are willing to pay for the network traffic generated by using the mobile application	60(42.9)	74(52.9)	5(3.6)	1(0.7)	0(0.0)
Satisfaction	You are satisfied with the mobile application overall	61(43.6)	74(52.9)	5(3.6)	0(0.0)	0(0.0)
	Feeling noticed through the services provided by this mobile application	76(54.9)	60(42.9)	4(2.9)	0(0.0)	0(0.0)

## Discussion

### The information-based education and training platform can reduce the incidence of WPV among nurses

The high incidence of WPV among nurses has exerted serious adverse effects on them. Educational training is a recommended intervention to help nurses cope with WPV, but further experiments are necessary to confirm its effectiveness. In this study, the overall rates of WPV exposure, verbal aggression, and verbal threat among nurses in both groups showed a trend of initially increasing and then decreasing 1 month after the intervention. On the other hand, the incidence of physical aggression showed a continuous decrease, likely due to the comprehensive coverage of WPV concepts in the training course, which improved the nurses' perception of violence. Cognition plays a crucial role in influencing nurses' judgment of whether they are experiencing violence. The study revealed that some nurses lacked sufficient knowledge about WPV, considering only physical aggression as a violent act while neglecting that verbal violence also falls within the category of WPV. Consequently, the actual reporting rate of verbal violence remains low [40–42]. A time effect was observed on the incidence of WPV in both groups in terms of overall suffering, verbal aggression, verbal threat, and physical aggression. This finding indicated that the incidence of WPV in both groups was gradually decreased over time, likely because the interventions included training on recognizing, preventing, and coping with WPV, leading to improved abilities in these areas among the nurses who received the training. These findings align with the results of several overseas studies [43–45]. A significant between-group effect was observed on the incidence of physical aggression among the two groups of nurses, with a greater decrease in the intervention group compared to the control group. Additionally, differences in the overall exposure rate and the incidence of verbal aggression were found at 3 and 6 months after the intervention, indicating that the online intervention group was more effective than the offline intervention group.

Compared with traditional training modes, the online information platform offers several advantages. It is not restricted by time or geographical location, is easy to operate, and allows nurses to access relevant courses as per their individual needs at any time and from anywhere. The platform is tailored to nurses' needs and offers content that is more suitable for the current education and training environment, making it more conducive to reducing the incidence rate of WPV. Wenjing Tu's study [46] has also shown that optimal results are achieved when the information support is aligned with the individual's current needs. Hence, in this study, the education and training courses were constructed based on the nurses' needs, making them more applicable to the nurse group.

The difference in the overall rate of WPV exposure, verbal aggression, verbal sexual harassment, and incidence of physical and sexual harassment before and 1 month after the intervention in the online intervention group was not statistically significant. This might be attributed to the nurses needing a transition period to become familiar with the rules of using the online intervention for the first time. However, statistically significant differences were observed in the overall incidence of WPV, verbal aggression, and verbal threat when comparing the pre-intervention and 3- and 6-month post-intervention periods for both groups of nurses. This highlighted the effectiveness of the online intervention in reducing the overall rate of WPV suffered by nurses, as well as the incidence of verbal aggression and verbal threat. Regarding verbal sexual harassment and physical, sexual harassment, the differences in incidence at different time points were not statistically significant. This might be due to nurses experiencing relatively few acts of sexual harassment in their clinical work, leading to a low number of applications of the strategies during the intervention period, making short-term effects difficult to observe. The study revealed that nurses experienced a high incidence of psychological violence, ranging from 56.85% to 86%, and a lower incidence of sexual violence, ranging from 3.3% to 11.5% [47–49]. Therefore, future interventions could be continued for a longer period of time in order to more effectively assess the effectiveness of the application. The nurses in the offline control group only showed a significant difference in WPV overall suffering and the incidence of verbal threat at 1 month and 6 months after the intervention. However, when comparing the incidence of physical aggression before and 6 months after the intervention, the difference was also statistically significant. This finding suggested that the offline intervention had difficulty achieving a sustained effect, possibly because the training content was extensive, making it challenging for nurses to consolidate and absorb all the information.

### The information-based education and training platform can reduce the severity of WPV suffered by nurses

Both between-group and time effects were observed for the severity of physical violence, along with a time effect for the severity of psychological violence in both groups of nurses. These findings indicated that both intervention methods successfully reduced the severity of violence suffered by nurses, with the intervention group showing greater effectiveness in reducing the severity of physical violence compared to the control group. The analysis suggested that offline training might be more challenging in simulating and rehearsing physical violence de-escalation strategies. As a result, nurses might find it difficult to understand and remember such strategies. On the other hand, the online platform allowed nurses to repeatedly pause and review content, making it easier for them to master and rehearse the de-escalation techniques effectively. Regarding the impact on the severity of psychological violence, the two types of training did not show statistically significant differences. This might be due to the nature of WPV, which is more inclined toward psychological violence. Additionally, due to varying awareness and understanding of aggression, along with cultural differences, verbal aggression may not have received enough attention in China. Nurses might focus more on the severity of physical violence and pay less attention to psychological trauma [50–52]. However, it is essential to recognize that violence in any form and at any level can have a lasting emotional impact on nurses and affect their psychological health. Severe violence may even lead to traumatic stress syndrome and result in serious burnout. Thus, addressing and reducing the severity of violence among nurses should be a significant concern [53–55].

### The information-based education and training platform can improve the level of WPV coping resources for nurses

WPV coping resources refer to the essential resources used to employ coping strategies in response to sources of violent stress. These resources include awareness of violence, foresight, coping ability, and social support [27]. Research has indicated that these coping resources act as a "buffer," and the greater the availability of coping resources, the less negative the consequences of WPV [38]. Therefore, increasing the level of coping resources is a crucial aspect of reducing the occurrence of WPV. At the baseline, the total coping resources score of nurses in the intervention group was 66.00 (59.00, 82.00), and in the control group, it was 66.00 (58.00, 77.00). These scores were higher than those reported by Peixi Wang [38] and Jianzheng Cai [37] in the previous 2 years. This increase might be attributed to hospitals gradually recognizing the impact of violence and actively educating nurses about it in recent years, consequently raising the overall level of nurses' coping resources. The study found significant between-group effects, time effects, and interaction effects for the total coping resources score and dimensions of nurses in both groups. This finding suggested that education and training could effectively improve nurses' coping resources level, and the platform intervention group experienced a more rapid increase in coping resources score. In a previous study [50], coping resources are found to be related to nurses' gender, age, and position. Therefore, when improving subsequent training content, it is essential to consider the differences among individuals, especially in enhancing coping resources for female medical staff.

### Information-based education and training platform is easy to operate and meets the needs of clinical nurses

In this study, the platform was evaluated from multiple perspectives, and the results showed that more than 95% of the nurses chose "strongly agree" or "agree" in terms of usefulness, ease of learning, acceptance, and satisfaction. This finding indicated that the education and training platform performed exceptionally well in terms of these aspects. The study [56–58] demonstrated that nurses' willingness to use mobile nursing applications was positively correlated with their perceived usefulness. The more useful the platform was perceived to be, the stronger their willingness to use it. Our developed prevention training platform was functional, simple, and easy to operate, enabling nurses to identify violent behaviors in advance while providing step-by-step coping strategies. According to the background statistics, within 6 months of the platform's operation, 140 nurses in the intervention group actively used the platform and completed all the questionnaires in the "Assessment and Feedback" module. The platform's total logging time was 12,230.7 h, with an average of 3.64 h per capita per week. It was accessed 87,360 times, with an average of 26 times per capita per week. Additionally, the "WPV reporting" module received a total of 187 nurse WPV reports, all of which were handled accordingly by managers. These results indicated that at the individual level, the information platform was convenient for clinical application, and nurses were more willing to use it with better compliance. At the organizational management level, the platform strengthened the follow-up treatment of violent incidents, facilitated regular tracking of the psychological and physical health status of violence victims, analyzed the types and characteristics of violent incidents, and contributed to a virtuous cycle of violence management in hospitals. The platform operated smoothly throughout the intervention period, without any operational failures, providing a positive user experience for nurses. However, in the trust dimension, 13.6% of nurses expressed concern about information leakage, and 12.9% felt that the hospital could not properly secure their information. This might be related to the information platform encouraging nurses to report WPV incidents. Studies by Yang Bin [59] and Alsmael et al. [60] have also shown that nurses often worry about criticism, punishment, or potential career implications after reporting violent incidents. Although our study did not yet thoroughly explore this phenomenon, future investigations can focus on analyzing the facilitators and deterrents that influence nurses' proactive reporting of violence. By understanding these factors, the platform's content can be improved and supplemented to increase the nursing staff's motivation to report.

## Conclusion

Despite the increasing use of informatics applications, there has been limited research focused on developing informatics applications specifically designed to address nurse WPV. Our study aimed to fill this research gap by describing the development and initial application of a nurse WPV informatics education and training platform. The goal was to assess the platform's effectiveness in reducing the incidence and severity of nurse WPV and improving coping resources. The results indicated that the WPV informatics education and training platform effectively met the demand for nurse WPV training. It successfully reduced the incidence of nurse WPV, minimized the severity of WPV suffered by nurses, increased nurses' willingness to report WPV incidents, and enhanced their coping resources related to WPV. Additionally, the platform introduced innovative approaches to hospital WPV management, demonstrating good practicality and feasibility. Overall, these findings suggested that the nurse WPV informatics education and training platform held great promise and was worthy of further promotion and implementation.

## Implication

This study verified the effectiveness of information-based education and training platform in reducing nurse WPV, and provided a reference for future education and training. Based on the research results, we suggest: (1) Actively promote the platform in other hospitals and verify the effectiveness of the platform in a large sample population, gradually modify and improve the platform in the process. (2) Provide adequate social support to nurses, and encourage nurses to report incidents of violence on the platform. We also suggest that the government and social media: (1) Improve WPV related laws and regulations, and severely crack down on WPV. (2) Prohibit the events of stigma toward medical staff and ease the relationship between medical staff and patients.

## Limitations

There are some limitations in our study that need to be addressed and improved through follow-up research. Firstly, our study population was limited to a few departments in one hospital, and there were certain cultural and structural barriers that might restrict the generalizability of the results to other settings. Secondly, although we used an information technology platform for education and training and observed positive improvements in the indicators, we noticed that the application effect diminished after 6 months when the intervention time was extended. In future research, extending the intervention time and thoroughly investigating the reasons for the decline in the application effect could be explored. Therefore, multi-center and larger sample size studies should be conducted in the future, while extending the intervention time to further improve and enhance the platform. We hope we can achieve more comprehensive and reliable findings, contributing to the advancement of knowledge and practical strategies in addressing nurse WPV.

## Data Availability

The data that support the findings of this study are available on request from the corresponding author, [initials], upon reasonable request.
